# Low autophagy capacity implicated in motor system vulnerability to mutant superoxide dismutase

**DOI:** 10.1186/s40478-016-0274-y

**Published:** 2016-01-25

**Authors:** Eiichi Tokuda, Thomas Brännström, Peter M. Andersen, Stefan L. Marklund

**Affiliations:** Department of Medical Biosciences, Umeå University, Building 6 M, 2nd Floor, Umeå, SE 901 85 Sweden; Department of Pharmacology and Clinical Neuroscience, Umeå University, Umeå, SE 901 85 Sweden; Present Address: Department of Chemistry, Keio University, 3-14-1, Hiyoshi, Yokohama, 223-0061 Kanagawa Japan

**Keywords:** Amyotrophic lateral sclerosis, Autophagy, Motor system vulnerability, Protein aggregates, Superoxide disumutase-1

## Abstract

**Introduction:**

The motor system is selectively vulnerable to mutations in the ubiquitously expressed aggregation-prone enzyme superoxide dismutase-1 (SOD1).

**Results:**

Autophagy clears aggregates, and factors involved in the process were analyzed in multiple areas of the CNS from human control subjects (*n* = 10) and amyotrophic lateral sclerosis (ALS) patients (*n* = 18) with or without *SOD1* mutations. In control subjects, the key regulatory protein Beclin 1 and downstream factors were remarkably scarce in spinal motor areas. In ALS patients, there was evidence of moderate autophagy activation and also dysregulation. These changes were largest in SOD1 mutation carriers. To explore consequences of low autophagy capacity, effects of a heterozygous deletion of Beclin 1 were examined in ALS mouse models expressing mutant SOD1s. This caused earlier SOD1 aggregation, onset of symptoms, motor neuron loss, and a markedly shortened survival. In contrast, the levels of soluble misfolded SOD1 species were reduced.

**Conclusions:**

The findings suggest that an inherent low autophagy capacity might cause the vulnerability of the motor system, and that SOD1 aggregation plays a crucial role in the pathogenesis.

**Electronic supplementary material:**

The online version of this article (doi:10.1186/s40478-016-0274-y) contains supplementary material, which is available to authorized users.

## Introduction

Amyotrophic lateral sclerosis (ALS) is an adult-onset neurodegenerative disorder characterized by loss of the upper and lower motor neurons. The disease begins focally and then spreads contiguously, resulting in progressive paralysis and finally death from respiratory failure [45]. Next to *C9orf72* [15], the most common known cause of ALS is mutations in the gene of the antioxidant enzyme superoxide dismutase-1 (*SOD1*), which are found in 2.5–6 % of the cases [1]. SOD1 is ubiquitously expressed, and the cause of the selective vulnerability of the motor system is not understood [30]. Over 180 mutations in SOD1 have been identified in ALS patients (http://alsod.iop.kcl.ac.uk/) [53]. They confer a cytotoxic gain of function to the enzyme, which is also poorly understood. Several of the mutations cause long C-terminal truncations or other disruptive changes in the expressed protein, precluding native folding. This suggests that any cytotoxicity mechanism that is common to the SOD1 mutants would originate from misfolded SOD1 species.

Inclusions containing aggregated SOD1 are hallmarks of ALS, both in patients and in transgenic animal models expressing mutant human SOD1s (hSOD1) [31]. This fits with a noxious role of hSOD1 un/misfolding, which allows formation of the non-native contacts present in aggregated species [34]. However, it is currently unknown whether the hSOD1 aggregation drives the pathogenesis of ALS, whether it is harmless, and even whether it represents protective scavenging of more toxic soluble misfolded species when the proteostasis is terminally compromised.

Macroautophagy, hereafter called autophagy, is the principal pathway by which cellular protein aggregates are cleared [12]. To gain further insight into the role of aggregation in ALS, we examined autophagy factors in several areas of the CNS in patients and in non-neurological controls and controls with neurodegeneration. We found remarkably low content of the key autophagy regulator Beclin 1 and downstream factors in the motor area of spinal ventral horns in the controls, and we also found evidence for activation of autophagy and dysregulation in ALS patients. To investigate the consequences of a low autophagy capacity, the effects of a heterozygous deletion of the *Becn1* gene were tested in hSOD1 transgenic mouse models with characteristics resembling ALS in humans. This caused earlier hSOD1 aggregation, onset of symptoms, and motor neuron loss, and also markedly shortened survival. However, there were reduced levels of soluble misfolded hSOD1 species, suggesting that aggregation could be the prime driver of neurotoxicity.

## Materials and methods

### Human subjects

The human study was performed according to the tenets of the Declaration of Helsinki. The collection of human tissues and their use was approved by the Swedish Medical Ethical Review Board. After informed consent from the relatives and whenever possible from the patients, specimens of CNS gray matter, including temporal lobe (ventral 3^rd^ of temporal superior gyrus), frontal lobe (anterior cingulate gyrus), cerebellar vermis, precentral gyrus (hemisphere close to the medial border), and spinal cord, were obtained at autopsy. The dissected segments of the spinal cord included the lamina IX of the ventral horn and the dorsal horn at the cervical or lumbar levels. Altogether, 28 human subjects were examined: five non-neurological controls (mean age at death: 60 ± 14 [SD] years, range 43–80 years), five Parkinson’s disease (PD) patients (77 ± 6 years, range 67–82) , four sporadic ALS (SALS) patients (70 ± 10 years, range 62–83), five familial ALS (FALS) patients without *SOD1* mutations (58 ± 8 years, range 49–68) including four patients with expanded hexanucleotide GGGGCC repeats in *C9orf72*, and nine FALS patients with *SOD1* mutations (60 ± 11 years, range 43–75) including one A4V [2], one G72C [48], five D90A [30], and two G127^ins*tggg*^ (G127X) [27, 28]. None of the five ALS patients without *SOD1* or *C9orf72* mutations carried ALS-associated mutations: all tested negative for a panel of ALS-associated genes including *ANG*, *FUS*, *OPTN*, *SQSTM1/p62*, *TARDBP*, *TBK1*, *UBQLN2*, or *VAPB*. All ALS patients fulfilled the revised El Escorial criteria for clinically definite or probable ALS [10]. The postmortem time for all patients varied between 0 and 3 days, and there were no systematic differences among the groups (Table 1).

**Table 1:** Information on humans subjects used in this study

Case	Diagnosis	Sex	Gene mutation	Age at death (years)	Postmortem delay (hours)
1	Controls	M	None	43	N.A.
2	Controls	F	None	80	48
3	Controls	F	None	64	42
4	Controls	M	None	55	48-72
5	Controls	M	None	58	29
6	SALS	F	None	69	44
7	SALS	F	None	83	48-72
8	SALS	F	None	62	52
9	SALS	F	None	64	24
10	FALS	M	C9orf72	49	23
11	FALS	M	Not found	64	48-72
12	FALS	M	C9orf72	52	20
13	FALS	F	C9orf72	55	13
14	FALS	M	C9orf72	68	50
15	FALS	F	SOD1 A4V	62	N.A.
16	FALS	F	SOD1 G72C	73	N.A.
17	FALS	M	SOD1 D90A	75	29
18	FALS	F	SOD1 D90A	64	50
19	FALS	M	SOD1 D90A	43	32
20	FALS	M	SOD1 D90A	53	11
21	FALS	M	SOD1 D90A	66	56
22	FALS	F	SOD1 G127X	62	20
23	FALS	M	SOD1 G127X	44	36-50
24	PD	M	N.A.	67	52
25	PD	F	N.A.	82	72-96
26	PD	F	N.A.	80	8
27	PD	M	N.A.	81	72
28	PD	F	N.A.	76	N.A.

*SALS* sporadic ALS, *FALS* familial ALS, *PD* Parkinson’s disease, *M* male, *F* female, *N.A.* not available

The CNS tissues were extracted as described previously [17, 30]. For analysis of the CNS expression patterns of autophagy factors, temporal lobe (ventral 3^rd^ of temporal superior gyrus) from a non-neurological control (case 4, Table 1) was used as a standard to allow multiple comparisons between different gels.

### Generation and analyses of ALS model mice with impaired autophagy

As mouse models of ALS, we used two distinct lines of transgenic mice carrying mutant human SOD1s: hSOD1^G127X^ [28] and hSOD1^G93A^ [23]. The hSOD1^G127X^ mice were generated in our laboratory (line 716), whereas the hSOD1^G93A^ mice were purchased from Jackson Laboratories (strain name: B6SJL-Tg(SOD1-G93A)1Gur/J; stock number: 002726). Both lines were back-crossed into a C57BL/6 J background for more than 30 generations.

The mouse strain with a heterozygous deletion of *Becn1* was kindly provided by Professor Beth Levine [44]. The *Becn1*^+/-^ mice were maintained in a CBA background. Male hSOD1^G127X^ or hSOD1^G93A^ mice were crossed with female *Becn1*^+/-^ mice. The genotype of the offspring was determined using PCR as described previously [44]. To avoid effects of different genetic backgrounds, non-transgenic and single-mutant mice from the litters were used for comparison with the double-mutant mice.

All animal procedures were carried out according to the guidelines of the Animal Care and Use Committee of the Umeå University. The animal protocols were approved by the Umeå Ethical Committee on Animal Experiments.

### Phenotyping of the mice

The disease courses of the mice were evaluated according to criteria based on changes in body weight [6]. Disease onset was regarded as the time when each mouse reached its peak weight. The endpoint was defined as the age at which a mouse was unable to right itself within 5 s after being pushed onto its side. The duration of disease was regarded as the period from onset of disease until the endpoint.

The hSOD1^G127X^/*Becn1*^*+/-*^ mice and their littermates were analyzed at three distinct stages of the disease: a presymptomatic stage (150 days), a symptomatic stage (10 % weight loss), and a terminal stage (*n* = 5 per genotype per disease stage). The hSOD1^G93A^/*Becn1*^*+/-*^ mice and their littermates were examined at a symptomatic stage (10 % weight loss) and a terminal stage (*n* = 4 per genotype per disease stage). The lumbar half of the spinal cord was immediately dissected, frozen in liquid nitrogen, and stored at −80 °C until use. The spinal cords of mice were extracted in phosphate-buffered saline (pH 7.0) containing 1 % (v/v) Nonidet P-40 and EDTA-free Complete® protease inhibitor cocktail (Roche Applied Science) as described previously [51], yielding detergent-soluble and insoluble fractions.

### Western immunoblot

Tissue extracts (20 μg protein) were electrophoresed on Criterion® TGX gels (Any kD; Bio-Rad), and were blotted onto polyvinylidene difluoride membranes (GE Healthcare). The blots were incubated with the following primary antibodies: anti-Beclin 1 (1:10,000; #3495; Cell Signaling Technology), anti-autophagy related gene (Atg) 12 (1:5,000; #4180; Cell Signaling Technology), anti- microtubule-associated protein light chain 3 (LC3) (1:10,000; PM036; Medical & Biological Laboratories), anti-p62 (1:2,000; 610832; BD Biosciences), anti-lysosome associated membrane protein 2 (Lamp2) (1:10,000; PA1-655; Thermo Scientific), anti-Cathepsin D (1:20,000; ab6313, Abcam), anti-glucose-regulated protein 78 kDa (Grp78) (1:10,000; NB100-91794; Norvus Biologicals), and anti-C/EBP homologous protein (Chop) (1:2,500; sc-575; Santa Cruz Biotechnology). β-Actin (1:50,000; MAB1501R; Millipore) was used as a loading control. For SOD1 analysis, we used anti-hSOD1^G127X^ (0.01 μg/mL) [28], anti-hSOD1 (0.001 μg/mL; human-specific, raised against a peptide corresponding to amino acids 24–39 of hSOD1) [17], or anti-murine SOD1 antibodies (0.1 μg/mL, raised against a peptide corresponding to amino acids 24–36 in murine SOD1) [29]. As secondary antibody, horseradish peroxidase-conjugated anti-rabbit or anti-mouse IgG (1:25,000; Dako) was used. The immunoreaction was visualized using an ECL Select reagent (GE Healthcare). The chemiluminescence of the blots was quantified using Quantity One software (Bio-Rad).

### Quantification of insoluble ubiquitinated proteins

Equal volumes of the detergent-insoluble fractions were separated on 4–15 % Criterion® TGX gels (Bio-Rad) and analyzed using western blot with anti-ubiquitin antibody (1:25,000; Z0458; Dako), which reacts with both Lys48- and Lys63-linked chains [50]. β-Actin in whole homogenates (1:50,000; MAB1501R; Millipore) was used as an internal marker.

### Analysis of hSOD1 aggregates using a filter trap assay

Amounts of large hSOD1 aggregates in the lumbar half of the spinal cords were quantified using a filter trap assay as described previously [5]. The following primary antibodies were used: anti-hSOD1^G127X^ (0.03 μg/mL) [28] and anti-hSOD1 (0.03 μg/ml; raised against a peptide corresponding to amino acids 57–72 of hSOD1) [17]. The aggregate standard (=100 %) was a homogenate of a spinal cord of a terminally ill hSOD1^G93A^ mouse kept in multiple aliquots in a freezer. The intensity of the staining of the tissue homogenates were related to staining of this standard keeping track of the dilutions used.

### Histopathology

Mice were perfused transcardially with saline followed by 4 % (w/v) paraformaldehyde in saline (pH 7.4). The lumbar spinal cords were harvested and embedded in paraffin. The lumbar sections (6 μm thickness) were immunostained with the following primary antibodies: anti-hSOD1^G127X^ (0.1 μg/mL) [28], anti- glial fibrillary acidic protein (GFAP) cocktail (0.01 μg/mL; 556330; BD Biosciences), and anti- ionized calcium-binding adapter molecule 1 (Iba1) (0.05 μg/mL; 019-19741; Wako Pure Chemicals). The sections were incubated with biotinylated secondary antibody against rabbit IgG or mouse IgG (1:200; Vector Laboratories). The immunoreaction was amplified using the VECTASTAIN® ABC Kit (Vector Laboratories) and was detected using 3,3’-diaminobenzidine (Dako) as the chromogen. The sections were imaged using a Pannoramic 250 Flash II scanner (3D Histech Ltd.).

Counting of α-motor neurons was performed as described previously [22], with slight revision. Serial transverse sections of the lumbar region were cut with a slice thickness of 6 μm. To avoid repeated counting of the same α-motor neuron, every tenth lumbar section of the spinal cord (L1–L3) was immunostained with anti-NeuN antibody (1 μg/mL; MAB377; Millipore), which does not recognize γ-motor neurons [19]. The size of the soma of NeuN-positive neurons was measured using Pannoramic Viewer software (3D Histech Ltd.). The number of α-motor neurons (>400 μm^2^) in the ventral horn was counted in 10 sections per mouse (*n* = 3–5 per genotype).

### Measurement of proteasome activities

The chymotrypsin-like, trypsin-like, and caspase-like activities of proteasome were measured as described elsewhere [49] using a Proteasome-Glo^™^ Cell-Based Assay Kit (Promega). Background non-specific peptidase activities were determined by adding proteasome inhibitors: bortezomib (0.1 μg/mL for chymotrypsin-like activity; Calbiochem) or AdaAhx_3_L_3_VS (30 μg/mL for trypsin-like and caspase-like activities; Calbiochem). The proteasome activities were calculated by subtracting the values with the inhibitors (non-specific peptidase) from the values without the inhibitors (total peptidase).

### Statistics

All data are given as mean ± SD. All statistical tests were performed with Statcel 3 software (OMS Publishing Inc.). Temporal changes in body weight of mice were analyzed using repeated-measures ANOVA. The disease onset and survival of mice were compared using Kaplan-Meier analysis with log-rank test. After validation of data normality and homoscedasticity, the disease duration was analyzed using a two-tailed Welch’s *t*-test. Multiple group comparisons were performed using one-way ANOVA followed by Tukey-Kramer *post-hoc* test. Statistical significance was defined as *P* < 0.05. The “*n*” values indicate numbers of individual humans or animals but not replicate measures of one sample. All biochemical and histopathological studies were replicated at least twice to validate the findings.

## Results

### Exceptionally low concentrations of autophagy factors in human spinal ventral horns

**Fig. 1:**
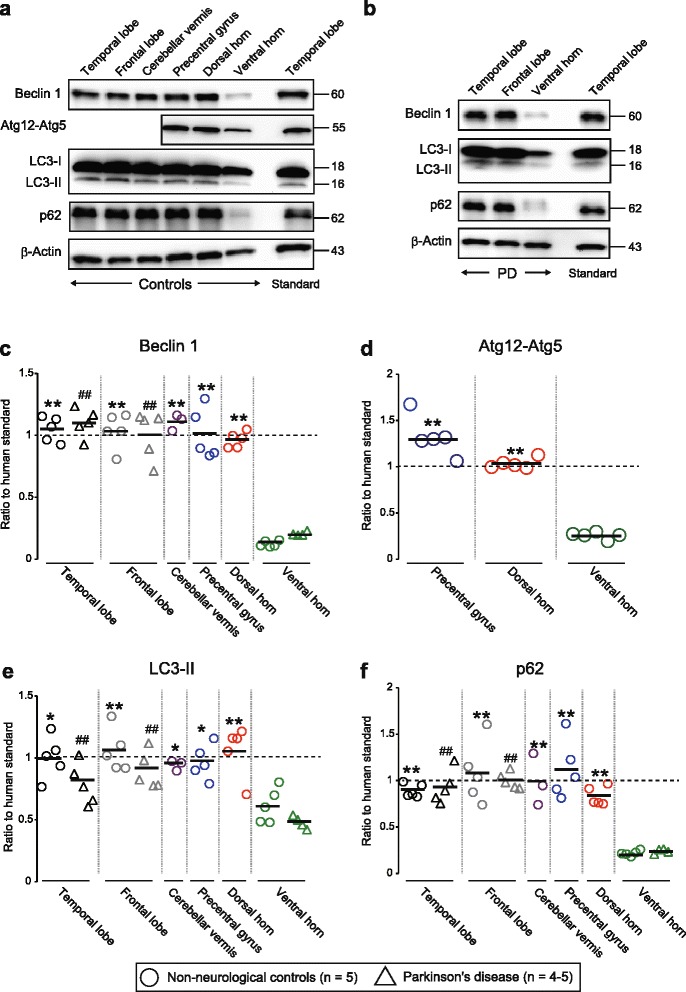
Autophagy factors are present in exceptionally low amounts in spinal ventral horns of human controls. **a**, **b** Western blots for autophagic proteins in human postmortem specimens of distinct CNS regions from (**a**) non-neurological and (**b**) neurodegenerative (Parkinson’s disease; PD) controls. Aliquots of protein extracts (20 μg) were loaded in each well. β-Actin was used as a loading marker. To allow multiple comparisons between different gels, grey matter from the anterior 3^rd^ of the temporal superior gyrus from a non-neurological control was used as a standard. Triplicate analyses of the proteins were made. Analysis of multiple triplicates indicated a relative standard deviation of the estimates of around 7 %. **c**–**f** Scatter plots showing the expression patterns of autophagic proteins including (**c**) Beclin 1, (**d**) Atg12 (detected as Atg12-Atg5 complex), (**e**) LC3-II, and (**f**) p62. All values in each CNS region were normalized to the level of expression of the standard. Bars represent mean values. *P < 0.05 vs. ventral horn of non-neurological controls. **P < 0.01 vs. ventral horn of non-neurological controls. ^##^ *P* < 0.01 vs. ventral horn of PD (one-way ANOVA with Tukey-Kramer’s test.)

Autophagy factors were analyzed in several areas of the CNS of controls and ALS patients with or without *SOD1* mutations. In non-neurological controls, Beclin 1, the principal initiator of autophagy [12], was found to be exceptionally low in lamina IX of the spinal ventral horns as compared to other gray-matter areas of the CNS (Fig. 1a, c). The ventral horn concentrations were equally low in PD patients, a neurodegenerative condition not affecting this area (Fig 1b, c). The levels of the downstream autophagy factors the Atg12-Atg5 complex, the LC3-II, and p62 were also low in the ventral horns of the controls (Fig. 1d, e, f), suggesting a low degree of basal autophagy activity.

**Fig. 2:**
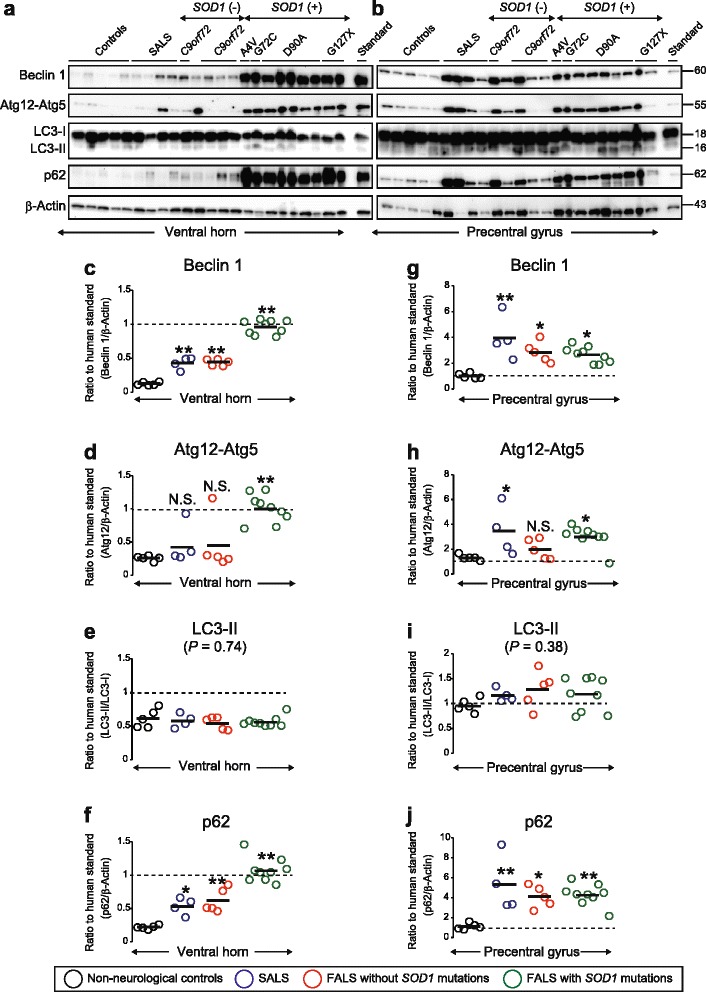
Autophagy factors become elevated in the spinal ventral horns and precentral gyrus of ALS patients. **a**, **b** Western blots for autophagic proteins in human postmortem specimens of (**a**) spinal ventral horns and (**b**) precentral gyrus from five non-neurological controls, four SALS patients, five FALS patients without *SOD1* mutations including expanded hexanucleotide GGGGCC repeats in *C9orf72* (*n* = 4), and nine FALS patients with *SOD1* mutations including one A4V, one G72C, five D90A, and two G127X. Aliquots of protein extracts (20 μg) were loaded in each well. β-Actin was used as a loading marker. **c**–**j** Scatter plots showing the expression levels of autophagic proteins in (**c**–**f**) ventral horn and (**g**–**j**) precentral gyrus. All values in each CNS region were normalized to the level of expression of the temporal lobe standard. Bars represent mean values. *P < 0.05 vs. controls. **P < 0.01 vs. controls. N.S. not significant (vs. controls). Statistical analysis was performed using one-way ANOVA with Tukey-Kramer’s test

In ALS patients, the levels of Beclin 1, Atg12-Atg5, and p62 were modestly elevated, more so in carriers of *SOD1* mutations than in apparently sporadic patients and carriers of other ALS-linked mutations (Fig. 2a, c, d, f). However, the concentrations only approached those found in other areas of the CNS in the control groups (Fig. 1). There were no changes in LC3-II (Fig. 2e). The primary motor cortex in the precentral gyrus, another area affected in ALS, also showed increases in Beclin 1 and p62 expression (Fig. 2b, g, j). The Atg12-Atg5 levels were significantly elevated in SALS and carriers of *SOD*1 mutations, but not in FALS without *SOD1* mutations (Fig. 2h). Again, no changes were found in LC3-II (Fig. 2i). Notably, in the case of the precentral gyrus, the changes were comparatively moderate in carriers of *SOD1* mutations.

The autophagy factors were similarly analyzed in spinal dorsal horns, temporal and frontal lobe gray matter, and cerebellar vermis, but in these areas there were no differences between the controls and the ALS groups (Additional file 1: Figure S1).

### Heterozygous deletion of *Becn1* in hSOD1^G127X^ mice exacerbated ALS-like disease

**Fig. 3:**
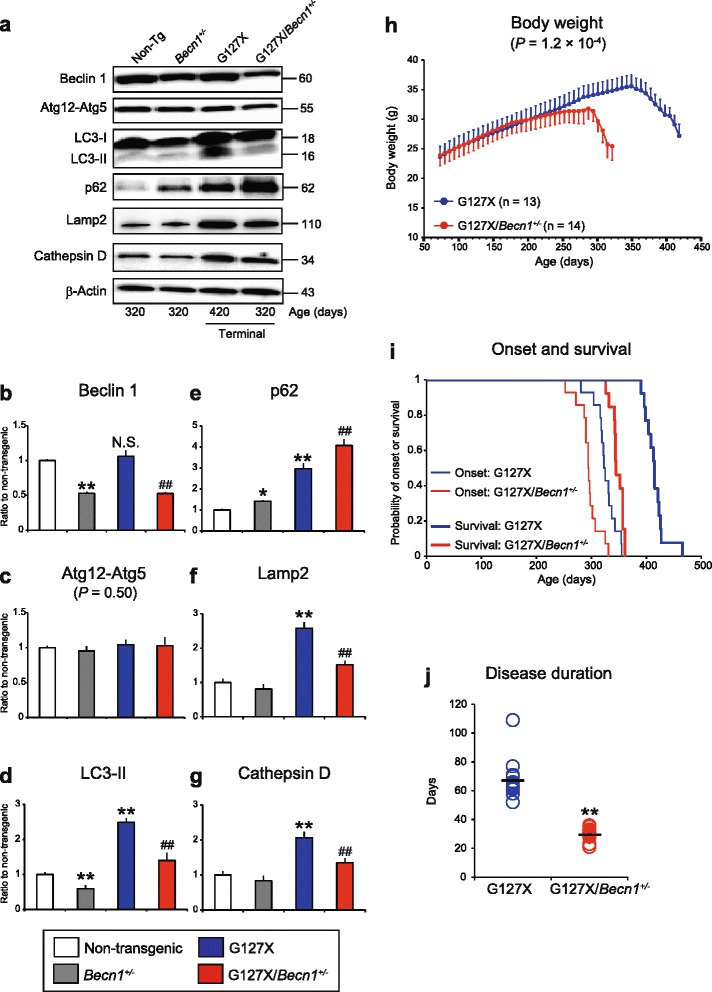
Heterozygous deletion of *Becn1* impairs autophagy and exacerbates the disease course in hSOD1^G127X^ mice. **a** Western blots for autophagic and lysosomal proteins in the spinal cords of hSOD1^G127X^/*Becn1* ^+/-^ mice and littermates (*n* = 3 per genotype). **b**–**g** The relative expression levels of (**b**) Beclin 1, (**c**) Atg12-Atg5, (**d**) LC3-II, (**e**) p62, (**f**) Lamp2, and (**g**) Cathepsin D in terminally ill mice. Data are given as mean ± SD. *P < 0.05 vs Non-Tg. **P < 0.01 vs Non-Tg. ^##^ *P* < 0.01 vs. hSOD1^G127X^ (one-way ANOVA with Tukey-Kramer’s test). N.S. not significant (vs. Non-Tg). **h**–**j** The disease courses of hSOD1^G127X^ mice (*n* = 13; male:female 7:6) and hSOD1^G127X^/*Becn1* ^+/-^ mice (*n* = 14; male:female 7:7) were evaluated based on alterations in body weight. **h** Temporal changes in body weight of the mice. Statistical significance was analyzed using repeated-measures ANOVA. **i** Kaplan-Meier curves for disease onset and survival. Statistical significance was analyzed using repeated-measures ANOVA using Kaplan-Meier analysis with log-rank test. **j** Scatter plot for the disease duration. Bars represent mean values. **P < 0.01 (two-tailed Welch’s *t*-test)

To explore the consequence of low autophagy capacity on hSOD1-induced motor neuron disease, the effects of heterozygous deletion of the *Becn1* gene were tested in mice expressing mutant hSOD1s. Homozygous deletion of *Becn1* leads to early embryonic lethality, whereas mice with a heterozygous deletion are essentially normal but show impaired autophagy [41, 44]. As our principal model, we chose mice expressing the truncated mutant hSOD1^G127X^ since it offers several advantages. All of this mutant hSOD1 exists as potentially toxic misfolded monomers in the CNS [54, 55]. The levels of such species can therefore be directly measured without confounding by the natively folded hSOD1 present in most other transgenic models [29, 54, 55]. Owing to rapid targeting for degradation, the hSOD1^G127X^ concentrations are low, less than one-half of those of the endogenous murine SOD1, minimizing the risk of overexpression artifacts [4, 28, 29]. Finally, mimicking ALS in humans, there is a long symptom-free period followed by a middle age onset and a relatively rapid disease course (Fig. 3i).

We verified that activation of autophagy occurred in hSOD1^G127X^ mice by analysis of autophagy and lysosome factors (Additional file 2: Figure S2). There were no changes in Beclin 1 and Atg12-Atg5, but there were significant increases in LC3-II, p62, Lamp2, and Cathepsin D already at 150 days, i.e. before onset of symptoms, and further increases at the symptomatic and terminal stages. This is similar to what has been found in other ALS models [25, 56]. In humans, there were also increases in Beclin-1 and Atg12-Atg5, but not LC3-II, suggesting the existence of some species differences in the autophagic response to SOD1-provoked ALS (Fig. 2c–f). Still, the results suggested that manipulation of autophagy in the mice might give information on the role of the system in aggregate clearance and on the neurotoxic role of the aggregates.

Male hSOD1^G127X^ mice were crossed with female mice with a heterozygous deletion of *Becn1*. In the resulting litters, as expected, the *Becn1* deletion halved the levels of Beclin 1 regardless of the hSOD1^G127X^ transgene (Fig. 3a, b). Although the level of Atg12-Atg5 was not altered (Fig. 3c), there was a 40 % reduction in LC3-II concentration and a 42 % increase in p62 concentration (Fig. 3d, e), suggesting that the reduction in Beclin 1 significantly impaired the autophagic flux in the spinal cords. Additionally, we found that the expression of lysosomal markers, Lamp2 and Cathepsin D, was increased in terminally ill hSOD1^G127X^ mice, but less so in hSOD1^G127X^/*Becn1*^+/-^ mice (Fig. 3f, g). A blocking effect of mutant hSOD1 on maturation of autophagolysosomes might conceivably also have contributed to the patterns observed.

The disease courses of the resulting hSOD1^G127X^/*Becn1*^+/-^ mice and hSOD1^G127X^ mice were monitored (Fig. 3h-j). Time of onset occurred 53 days earlier in hSOD1^G127X^/*Becn1*^+/-^ mice than in hSOD1^G127X^ mice (295 ± 19 vs. 348 ± 13 days; *P* = 5.1 × 10^-6^) (Fig. 3i). The disease duration was 38 days shorter (30 ± 4.4 days vs. 68 ± 13 days; *P* = 7.1 × 10^-10^) (Fig. 3j), and the hSOD1^G127X^/*Becn1*^+/-^ mice reached the end stage 90 days earlier than the hSOD1^G127X^ mice (326 ± 19 days vs. 416 ± 19 days; *P* = 3.0 × 10^-6^) (Fig. 3i).

**Fig. 4:**
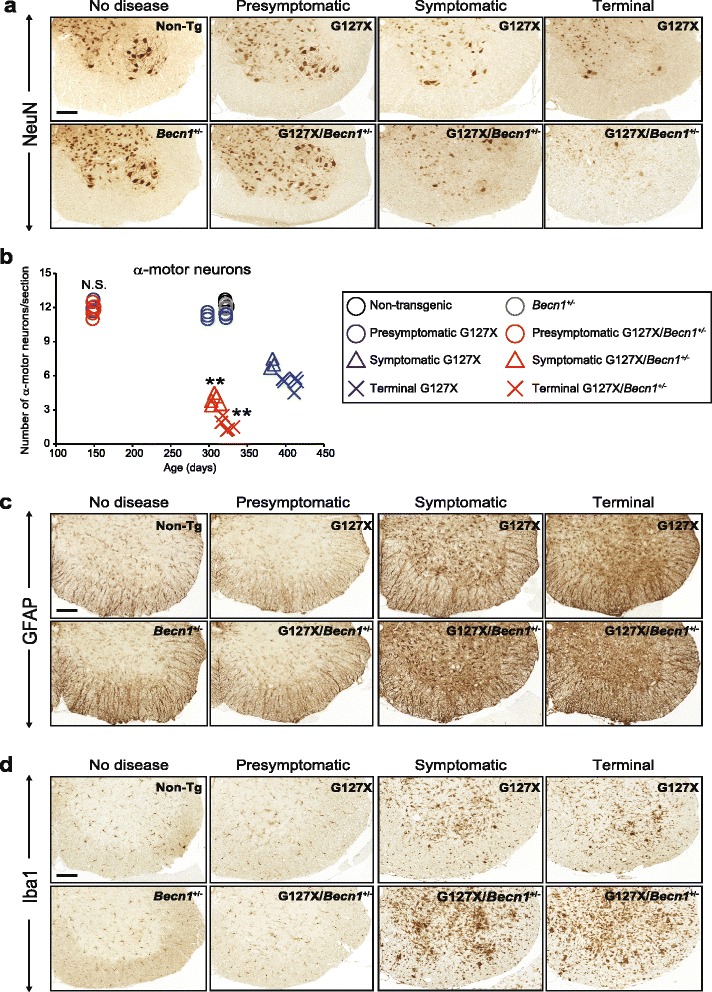
Impaired autophagy worsens ALS-related pathological changes in spinal cords of hSOD1^G127X^ mice. The hSOD1^G127X^/*Becn1* ^+/-^ mice and littermates were analyzed at three distinct stages of the disease: a presymptomatic (150 days), a symptomatic (10 % weight loss), and the terminal stage. The lumbar spinal cord sections were immunostained with antibodies against (**a**) NeuN, (**c**) GFAP, or (**d**) Iba1, which are specific markers for neurons, astrocytes, or microglia, respectively. We used 320-day-old non-transgenic (Non-Tg) and *Becn1* ^+/-^ mice. **b** Quantification of NeuN-positive α-motor neurons in the lumbar spinal cords (*n* = 3–5 per genotype). **P < 0.01 vs. disease stage-matched hSOD1^G127X^. N.S. not significant vs. 150-day-old hSOD1^G127X^ (one-way ANOVA with Tukey-Kramer *post-hoc* test.). Scale bars: 100 μm

Motor neurons were counted throughout the course of disease (Fig. 4a, b). Earlier and more extensive loss was found in hSOD1^G127X^/*Becn1*^+/-^ mice than in hSOD1^G127X^ mice at the symptomatic stage (43 ± 3.3 % in hSOD1^G127X^ vs. 70 ± 3.8 % in hSOD1^G127X/^*Becn1*^+/-^, *P* < 0.01) and at the terminal stage (57 ± 4.6 % vs. 87 ± 4.3 %, *P* < 0.01). In non-transgenic and *Becn1*^+/-^ mice, no changes in motor neuron count were seen. The more extensive motor neuron loss in hSOD1^G127X^/*Becn1*^+/-^ mice was accompanied by enhanced numbers and activation of astrocytes and microglia at the symptomatic and terminal phases (Fig. 4c, d).

Thus, deletion of even a single allele of *Becn1* in hSOD1^G127X^ mice results in exacerbation of the disease course―including onset, duration, lifespan, and loss of spinal motor neurons.

Since Beclin 1 is a haplo-insufficient tumor suppressor gene, *Becn1*^+/-^ mice develop tumors in several tissues including the lungs and liver when over 400 days old. These can be detected even by gross morphology [44]. To exclude the possibility that exacerbation of the disease course in hSOD1^G127X^/*Becn1*^+/-^ mice was associated with development of tumors, we examined the gross appearance of lungs and liver from terminally ill hSOD1^G127X^/*Becn1*^+/-^ mice, age-matched *Becn1*^+/-^ mice and non-transgenic mice (Additional file 3: Figure S3a). No changes were found. The rise in body weight in *Becn1*^+/-^ mice (*n* = 8; male:female 4:4) was essentially identical to that in non-transgenic mice (*n* = 10; male:female 4:6), at least until 320 days of age (*P* = 0.56), which was the mean lifespan of hSOD1^G127X^/*Becn1*^+/-^ mice (Additional file 3: Figure S3b).

### Heterozygous deletion of *Becn1* in hSOD1^G127X^ mice increased aggregated and decreased soluble mutant hSOD1

**Fig. 5:**
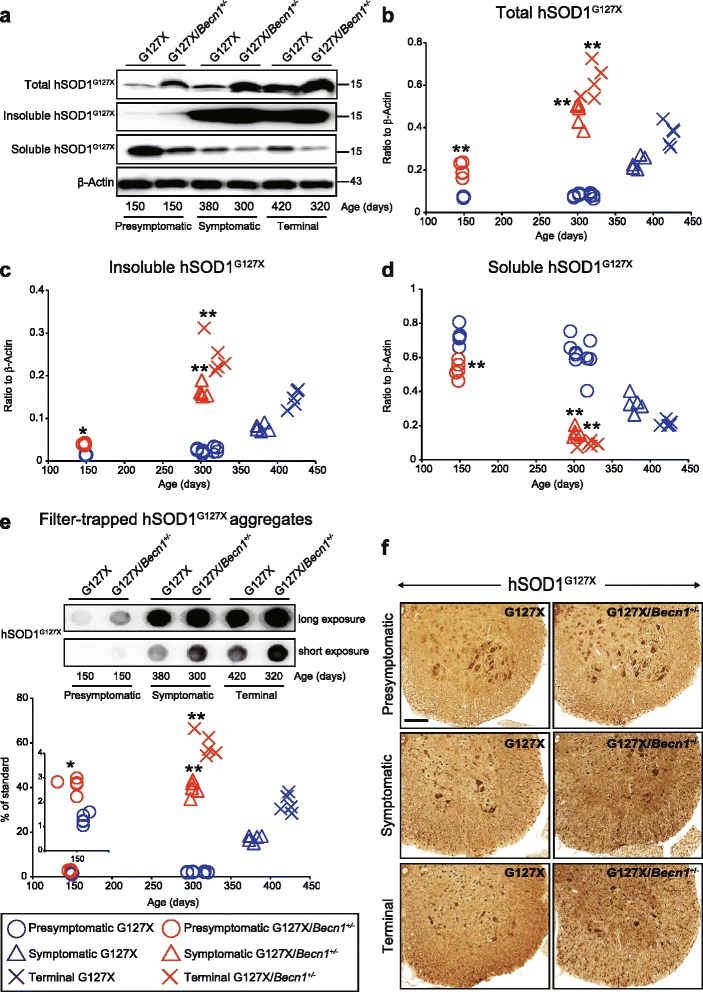
Impaired autophagy increases hSOD1^G127X^ aggregation, and reduces the amount of soluble species throughout the disease course. The spinal cords (*n* = 5 per genotype per disease stage) were separated into three distinct fractions based on detergent solubility: whole homogenates, detergent-insoluble, and detergent-soluble fraction. **a** Western blots for hSOD1^G127X^ protein in the three fractions. β-Actin in whole homogenates was used as a loading control. **b-d** Amounts of (**b**) total, (**c**) insoluble, and (**d**) soluble hSOD1^G127X^ protein. *P < 0.05 vs. hSOD1^G127X^ mice at 150 days of age. **P < 0.01 vs. disease stage-matched hSOD1^G127X^ mice (one-way ANOVA with Tukey-Kramer’s test). **e** *Upper*: Filter-trapped hSOD1^G127X^ aggregates in the spinal cords of the mice (n = 5 per genotype per disease stage). *Lower*: Amounts of hSOD1^G127X^ aggregates. *P < 0.05 vs. hSOD1^G127X^ mice at 150 days of age. **P < 0.01 vs. disease stage-matched hSOD1^G127X^ mice (one-way ANOVA with Tukey-Kramer’s test). **f** Immunohistochemistry for hSOD1^G127X^ protein in lumbar spinal cords of mice. Scale bars: 100 μm

The total concentration of hSOD1^G127X^ in spinal cords rose with time; at all ages, it was higher in hSOD1^G127X^/*Becn1*^+/-^ mice than in hSOD1^G127X^ mice (Fig. 5a, b). This was caused by increased levels of detergent-insoluble hSOD1^G127X^ (Fig. 5c), which were higher in hSOD1^G127X^/*Becn1*^+/-^ mice than in hSOD1^G127X^ mice. Even when compared at the same disease stages, the concentrations were higher in hSOD1^G127X^/*Becn1*^+/-^ mice: 2.0-fold and 1.6-fold at the symptomatic and terminal stages, respectively. In contrast, the concentrations of soluble hSOD1^G127X^ declined with time and at all stages they were lower in hSOD1^G127X^/*Becn1*^+/-^ mice than in hSOD1^G127X^ mice (Fig. 5d). As an independent method for analysis of hSOD1 aggregates, the spinal cord homogenates were analyzed with a dot-blot filter assay (Fig. 5e). The time-course patterns were very similar to those of the detergent-insoluble hSOD1^G127X^ species.

The hSOD1^G127X^ mutant in lumbar spinal cords was also examined by immunohistochemistry (Fig. 5f). At the presymptomatic stage, the highest levels of the protein were seen in the vulnerable α-motor neurons of the ventral horn. Other cell populations were more faintly stained. At the symptomatic and terminal stages, punctate aggregates also accumulated in the neuropil. The levels were clearly higher in the hSOD1^G127X^/*Becn1*^+/-^ mice.

To test the possibility that the drastic alterations in hSOD1^G127X^ fractions might to any degree be caused by changes in SOD1 synthesis in the compromised spinal cord, the endogenous murine SOD1 was analyzed with a mouse-specific antibody (Additional file 4: Figure S4). No changes in concentration were found and it was soluble: detergent-insoluble/aggregated murine SOD1 could not be demonstrated at any of the disease stages. This is in accord with previous studies showing that human and murine SOD1s do not coaggregate [5, 21].

### Equal degrees of ER stress in terminal hSOD1^G127X^ mice and hSOD1^G127X^/*Becn1*^+/-^ mice

Endoplasmic reticulum (ER) stress, a condition in which misfolded proteins accumulate in the lumen of the ER, has been implicated in the pathogenesis of mutant hSOD1 mouse models and ALS patients [3]. We analyzed the ER stress markers Grp78 and Chop, and found increases in terminally ill mice (Additional file 5: Figure S5). The final degree of activation was equal, but the stage analyzed appeared earlier in hSOD1^G127X^/*Becn1*^+/-^ mice than in hSOD1^G127X^ mice.

### Exacerbated disease also in hSOD1^G93A^/*Becn1*^*+/-*^ mice

**Fig. 6:**
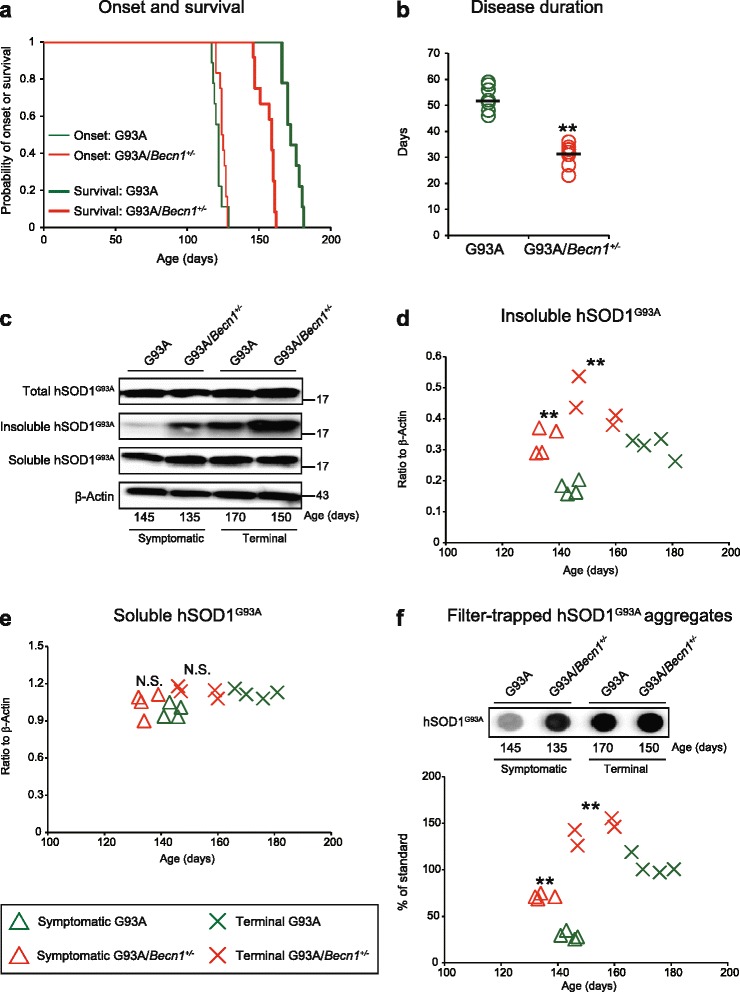
Heterozygous deletion of *Becn1* in hSOD1^G93A^ mice enhances aggregation and shortens survival. **a** Kaplan-Meier curves for disease onset and survival of hSOD1^G93A^ (*n* = 9; male:female 5:4) and hSOD1^G93A^/*Becn1* ^+/-^ mice (*n* = 12; male:female 7:5). **b** Scatter plot for disease duration. Bars represents the mean values. **P < 0.01 (two-tailed Welch’s *t*-test). **c** Western blots for hSOD1^G93A^ in whole homogenates, detergent-insoluble, and detergent-soluble fractions extracted from the spinal cords (*n* = 4 per genotype per disease stage). β-Actin in whole homogenates was used as a loading control. Amounts of (**d**) insoluble and (**e**) soluble hSOD1^G93A^ protein. **P < 0.01 vs. disease stage-matched hSOD1^G93A^ mice; N.S.: not significant vs. disease stage-matched hSOD1^G93A^ (one-way ANOVA with Tukey-Kramer’s test). **f** *Upper*: Filter-trapped hSOD1^G93A^ aggregates in the spinal cords of the mice (*n* = 4 per genotype per disease stage). *Lower*: Amounts of hSOD1^G93A^ aggregates. **P < 0.01 vs. disease stage-matched hSOD1^G93A^ (one-way ANOVA with Tukey-Kramer’s test)

Mice that express G93A mutant hSOD1 are the most commonly examined murine ALS model [23]. The model is aggressive, with early onset of symptoms and a short lifespan. The concentration of mutant hSOD1 is 17-fold higher than that of the endogenous murine SOD1, causing overexpression artifacts [4, 29]. Moreover, over 95 % of the protein is natively folded, making analysis of misfolded hSOD1 conformers complicated [54, 55]. For reference purposes, we performed a limited study of the effects of *Becn1* deletion using this model (Fig. 6).

The time of onset was not significantly different in hSOD1^G93A^ mice and hSOD1^G93A^/*Becn1*^+/-^ mice (121 ± 3.6 days vs. 125 ± 2.7 days; *P* = 0.23; Fig. 6a). However, the disease duration was much shorter (52 ± 4.8 days vs. 31 ± 4.6 days; *P* = 5.7 × 10^-9^; Fig. 6b), leading to shortened survival (173 ± 5.7 days vs. 156 ± 4.6 days; *P* = 2.0 × 10^-4^; Fig. 6a).

As with the hSOD1^G127X^ model, the amount of aggregated hSOD1 was higher in hSOD1^G93A^/*Becn1*^+/-^ mice than in hSOD1^G93A^ mice at both the symptomatic stage and the terminal stage (Fig. 6c, d, f). In contrast to the hSOD1^G127X^ mice, no changes could be discerned in the huge amounts of total and soluble hSOD1^G93A^ mutant present in the spinal cords (Fig. 6e).

Other changes were generally comparable to those in hSOD1^G127X^/*Becn1*^+/-^ mice, including autophagy factors, lysosomal markers, murine SOD1 expression, and ER stress markers (Additional file 4: Figure S4, Additional file 5: Figure S5 and Additional file 6: Figure S6).

### Larger increases in ubiquitinated insoluble proteins in hSOD1^G93A^ mice than in hSOD1^G127X^ mice

**Fig. 7:**
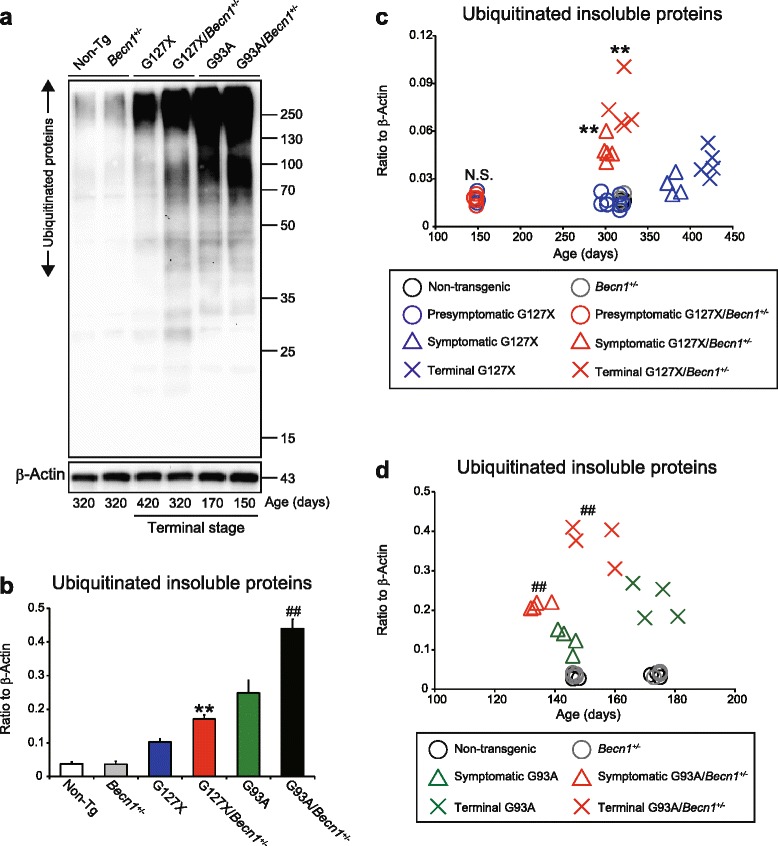
More accumulation of insoluble ubiquitinated proteins in hSOD1^G93A^ than hSOD1^G127X^ mice. **a** Western blots for ubiquitinated proteins in detergent-insoluble fractions extracted from the spinal cords of terminally ill mice and their littermates (*n* = 3 per genotype). β-Actin in whole homogenate was used as an internal marker. **b** Densitometric calculations of the relative expression levels of ubiquitinated insoluble proteins in various mouse genotypes. Data are given as mean ± SD. **P < 0.01 vs. hSOD1^G127X^. ^##^ *P* < 0.01 vs. hSOD1^G127X^/*Becn1* ^+/-^ (one-way ANOVA with Tukey-Kramer’s test). Temporal changes of ubiquitinated insoluble proteins in either (**c**) hSOD1^G127X^/*Becn1* ^+/-^ mice and littermates (*n* = 3–5 per genotype) or (**d**) hSOD1^G93A^/*Becn1* ^+/-^ mice and littermates (*n* = 4 per genotype). **P < 0.01 vs. disease stage-matched hSOD1^G127X^. ^##^ *P* < 0.01 vs. disease stage-matched hSOD1^G93A^. N.S. not significant vs. 150-day-old hSOD1^G127X^ (one-way ANOVA with Tukey-Kramer’s test)

The proteasome is the main pathway for degradation of ubiquitinated proteins. However, autophagy also has a role in the clearance of such proteins [46]. We thus quantified the amounts of ubiquitinated insoluble proteins (Fig. 7). The heterozygous deletion of *Becn1* did not in itself increase the concentration. In terminally ill hSOD1^G127X^ and hSOD1^G93A^ mice, the levels were significantly increased, and these increases were enhanced by the *Becn1* deletion (Fig. 7c, d). Remarkably, irrespective of Beclin 1 status, the increases were more than double as high in hSOD1^G93A^ mice as in hSOD1^G127X^ mice (Fig. 7a, b). The increases are not explained by impairments of the proteolytic activities of the proteasome (Additional file 7: Figure S7).

## Discussion

A main finding in the present study was the low Beclin 1 concentration in lamina IX of the spinal ventral horns from the two control groups. The other gray matter areas examined, including the dorsal horns, contained around seven times more Beclin 1 and, overall, they were similar to each other. In accord with the low Beclin 1 levels, there were also remarkably low concentrations of the downstream autophagy factors the Atg12-Atg5 complex, LC3-II and p62. The motor neuron demise caused by mutant SOD1 is partly non-cell autonomous since it is influenced by the presence of the mutant protein also in the surrounding glia [26]. Motor neuron somata and neurites have been estimated to occupy 20 % of the volume of the ventral horns [11], and together with supporting glia, they should account for a major proportion of the specimens analyzed here. Thus, low levels of autophagy factors should be a characteristic of the cells that form the spinal motor system. The cortical upper motor neurons are sparse in the gray matter of the precentral gyrus, and they would occupy a minor proportion of the volume. Thus, we cannot draw any firm conclusions regarding the autophagy capacity of the cells that form the upper motor system.

In the ALS patients, there was evidence of some activation of autophagy, both in the ventral horns and in the precentral gyrus. Notably, the subgroup carrying SOD1 mutations showed the greatest changes in the spinal ventral horns, whereas the increases in the precentral gyrus were more comparable in all ALS forms. This is in accord with the lower motor neuron emphasis in ALS patients with SOD1 mutations [2]. Despite the increases in the autophagy initiator Beclin 1 and the Atg12-Atg5 complex, there was no evidence of autophagy activation in terms of LC3-II elevations. Furthermore, the adaptor protein p62, which is degraded together with protein aggregates in autolysosomes [32], was accumulated, suggesting that the autophagic flux might be impeded in ALS. Thus, our findings might indicate a degree of autophagy dysfunction, particularly in mutant SOD1-induced ALS.

Inclusions containing misfolded SOD1 are found in not only patients carrying *SOD1* mutations, but also in both sporadic and familial cases that carry other ALS-linked mutant proteins [8, 16, 17, 21]. This suggests that the protein may be more generally involved in ALS. In this regard, it is noteworthy that the granular SOD1 inclusions within motor neurons of SALS patients colocalized partially with lysosomal markers [17], which supports the present evidence of dysregulation of autophagy in the disease.

We found here that a moderate reduction in autophagy by *Becn1*^+/-^ caused more aggressive disease in hSOD1^G127X^ ALS model mice. There was earlier onset, faster progression, and a markedly shortened lifespan. In accord with the accelerated disease course, there was also an earlier and more extensive loss of motor neurons. It is notable that in the terminal stage the loss was greater in the double mutant mice than in the hSOD1^G127X^ mice. It has been found that paralysis can be caused by both loss of motor neurons and dysfunction of remaining motor neurons [20]. Perhaps the latter mechanism played a proportionally greater role in the hSOD1^G127X^ mice. In parallel, an enhanced aggregation of hSOD1^G127X^ was found, showing the importance of autophagy in elimination of protein aggregates. In contrast, the concentration of soluble hSOD1^G127X^, which exists entirely as misfolded monomers [54, 55], was lower from the presymptomatic stage to the end stage. This is not explained by alterations in the proteasome, which is the primary system for degradation of misfolded proteins: the activity did not change during the course of the disease, and did not differ between the two mouse types. Nor do the alterations seem to stem from any changes in synthesis of SOD1 in compromised spinal cord: the concentration of the endogenous murine SOD1 was constant throughout the course of disease. Autophagy is thought to be responsible for turnover of long-lived proteins in the cytosol, but it apparently has no significant role in degradation of murine SOD1, which has a comparatively long half-life of 20 days in the CNS [42]. We propose that the lower concentrations of soluble misfolded hSOD1^G127X^ in the hSOD1^G127X^/*Becn1*^+/-^ mice result from high rates of recruitment into free fibril edges in the abundant hSOD1 aggregates (this is discussed in supplementary data in [35]). The findings are compatible with the notion that soluble, misfolded SOD1 species provoke ALS through formation of aggregates rather than through direct effects on critical components in motor-area cells. Whether this conclusion would hold for all ALS-linked SOD1 mutants is unknown, but in our view it is more likely that they all cause the disease by the same basic mechanism rather than through several individual ones.

Beclin 1 exerts its role in autophagy induction by being a membrane-bound scaffold which enables the recruitment of other autophagy proteins involved in nucleation and maturation of the autophagosome [12]. By binding to various specific proteins, Beclin can also localize to and regulate other vesicle trafficking pathways [37]. Weakening of these may have contributed to the phenotypes observed in the *Becn1*^+/-^ mice. Retromer trafficking is impaired in such mice leading to reduced phagocytosis of extracellular aggregates by microglia [36]. This may have contributed to the greater aggregate accumulation in the hSOD1 transgenic mice with the heterozygous *Becn1* deletion than in those without when compared at the same disease stages. Via binding to the UV radiation resistance-associated gene protein (UVRAG), Beclin 1 can localize to and regulate various endocytic pathways, and this function is important for the integrity of neurons [38]. Conditional *Becn1* knockout in neurons leads to impairment of endocytic pathways and accelerated neurodegeneration [38]. The degree of such impairment in mice with a heterozygous *Becn1* deletion is not known, but we cannot exclude some role in the exacerbated phenotypes of the present double mutant mice.

A further potential confounding mechanism is the binding of Beclin-1 to the antiapoptotic factors Bcl-2 and Bcl-X_L_ in the ER. Beclin 1 is inactive when present in such complexes and to exert its activities it has to be released, which can be accomplished by a variety of mechanisms [40]. The complex formation does, however, not seem to interfere with the activity of the antiapoptotic factors, and there is no convincing evidence that Beclin 1 can serve as a proapoptotic factor [13, 47]. Thus, it would appear less likely that any reduced cross-talk with the apoptotic system influenced the disease in the hSOD1^G127X^/*Becn1*^+/-^ mice.

The results in the hSOD1^G127X^/*Becn1*^+/-^ mice were corroborated in the more aggressive hSOD1^G93A^ model, although the effects of the heterozygous *Becn1* deletion were smaller. The *Becn1* deletion was even recently reported to amend the disease in mice expressing G86R mutant murine SOD1 [39]. Curiously, there was a non-significant delay in onset of symptoms, but a shortened symptomatic phase, together resulting in a 14-day prolongation of survival. Perhaps the *Becn1* deletion by some mechanism delayed the initiation of the murine SOD1^G86R^ aggregation, but still reduced the clearance of aggregates once the process was under way. The unexpected result may also be related to the fact that this transgenic model is even more aggressive than the hSOD1^G93A^ model. We found much greater increases in ubiquitinated insoluble proteins in the hSOD1^G93A^ model than in the hSOD1^G127X^ model. Part of the increases might be related to increased accumulation p62 owing to autophagy inhibition. Such increased p62 has been shown to inhibit delivery of substrate to the proteasome [33]. The increases in p62 were, however, comparable in the hSOD1^G93A^ and hSOD1^G127X^ models. Perhaps a greater load of aggregated proteins in the highly aggressive models leads to insufficiency in autolysosomal clearance [7]. In that situation, a high degree of autophagy flux might have negative effects, e.g. by enhancing lysosomal membrane destabilization [9]. Other explanations for the discrepancy could be that the SOD1^G86R^ mutant was murine and that it also affected another segment of the SOD1 protein. Thus, it cannot be excluded that the consequences of altering Beclin-1 levels and autophagic capacity may differ between both mouse models and different ALS patients.

The current findings may also shed light on the vulnerability of the motor system to mutations in other ubiquitously expressed proteins. Thus, mutations in *VCP*, *OPTN*, *UBQLN2*, *CHMP2B*, *SQSTM1/p62*, *DCTN1* [43], *TBK1* [14, 18] have all been suggested to cause impairment of the autophagy-lysosomal system [24]. Mutations in *TARDBP*, *FUS*, and *C9orf72* are all associated with potentially toxic protein aggregation [24]. Although the anatomical conditions only allowed assessment of the spinal motor system, the inherent low autophagy activity detected might contribute to the selective vulnerability both to aggregation-prone proteins and to mutations that further depress the autophagy-lysosomal pathway.

## Conclusions

The results of the study suggest that an inherent low autophagy capacity might cause the selective vulnerability of the motor system to mutant SOD1s, and that aggregation of the protein plays a crucial role in the pathogenesis. The findings may also explain the vulnerability to mutations in other proteins expected to weaken the autophagy-lysosomal pathway. This suggests that measures aimed at enhancing this pathway might be beneficial in treatment of ALS. The interventions which have been tested so far in preclinical systems and in patients have, however, yielded ambiguous results – perhaps calling for development of new concepts [52].

## Additional files

Additional file 1: Figure S1. No alterations in autophagic status in spinal dorsal horn, temporal lobe, frontal lobe, and cerebellar vermis from ALS patients. (EPS 1998 kb) Additional file 2: Figure S2. Alterations in autophagy and lysosome factors in spinal cords of hSOD1^G127X^ mice over the course of disease. (EPS 2037 kb) Additional file 3: Figure S3. Exacerbation of the disease course in hSOD1^G127X^/*Becn1* ^*+/*-^ mice does not result from the development of tumors. (EPS 14820 kb) Additional file 4: Figure S4. Impaired autophagy does not influence the amounts of murine SOD1. (EPS 2296 kb) Additional file 5: Figure S5. Equal degrees of ER stress in terminal mutant hSOD1 mice irrespective of Beclin 1 status. (EPS 2004 kb) Additional file 6: Figure S6. Heterozygous deletion of *Becn1* impairs autophagy in hSOD1^G93A^ mice. (EPS 2220 kb) Additional file 7: Figure S7. The proteolytic function of the proteasome is not impaired in in *Becn1* ^*+/-*^ mice carrying hSOD1 mutants. (EPS 1730 kb)

## Abbreviations

ALS: Amyotrophic lateral sclerosis
Atg: Autophagy related protein
Chop: C/EBP homologous protein
ER: Endoplasmic reticulum
FALS: Familial ALS
GFPA: Glial fibrillary acidic protein
Grp78: Glucose-regulated protein 78 kDa
G127X: Gly127^ins*tggg*^
hSOD1: Human SOD1
Iba1: Ionized calcium-binding adapter molecule 1
Lamp2: Lysosome associated membrane protein 2
LC3: Microtubule-associated protein light chain 3
PD: Parkinson’s disease
SALS: Sporadic ALS
SOD1: Superoxide dismutase-1
